# Multi-Year Lags between Forest Browning and Soil Respiration at High Northern Latitudes

**DOI:** 10.1371/journal.pone.0050441

**Published:** 2012-11-26

**Authors:** Ben Bond-Lamberty, Andrew G. Bunn, Allison M. Thomson

**Affiliations:** 1 Pacific Northwest National Laboratory, Joint Global Change Research Institute at the University of Maryland–College Park, College Park, Maryland, United States of America; 2 Department of Environmental Sciences, Huxley College, Western Washington University, Bellingham, Washington, United States of America; The Ohio State University, United States of America

## Abstract

High-latitude northern ecosystems are experiencing rapid climate changes, and represent a large potential climate feedback because of their high soil carbon densities and shifting disturbance regimes. A significant carbon flow from these ecosystems is soil respiration (*R*_S_, the flow of carbon dioxide, generated by plant roots and soil fauna, from the soil surface to atmosphere), and any change in the high-latitude carbon cycle might thus be reflected in *R*_S_ observed in the field. This study used two variants of a machine-learning algorithm and least squares regression to examine how remotely-sensed canopy greenness (NDVI), climate, and other variables are coupled to annual *R*_S_ based on 105 observations from 64 circumpolar sites in a global database. The addition of NDVI roughly doubled model performance, with the best-performing models explaining ∼62% of observed *R*_S_ variability. We show that early-summer NDVI from previous years is generally the best single predictor of *R*_S_, and is better than current-year temperature or moisture. This implies significant temporal lags between these variables, with multi-year carbon pools exerting large-scale effects. Areas of decreasing *R*_S_ are spatially correlated with browning boreal forests and warmer temperatures, particularly in western North America. We suggest that total circumpolar *R*_S_ may have slowed by ∼5% over the last decade, depressed by forest stress and mortality, which in turn decrease *R*_S_. Arctic tundra may exhibit a significantly different response, but few data are available with which to test this. Combining large-scale remote observations and small-scale field measurements, as done here, has the potential to allow inferences about the temporal and spatial complexity of the large-scale response of northern ecosystems to changing climate.

## Introduction

Climate changes in the coming century may affect permafrost thaw rates, greenhouse gas fluxes, wildfires, productivity, biota, and energy fluxes in northern ecosystems [1], [2], [3], [4]. Such high-latitude ecosystems represent a large potential climate feedback [5], [6] because of their high soil carbon densities [7] and rapid warming [8]. Any current or future carbon losses from these areas will mostly occur through combustion [9] or changes in the balance between net primary production and the heterotrophic component of *R*_S_, the soil surface CO_2_ flux between the soil and atmosphere. At 80–100 Pg C yr^−1^ [10], [11], total *R*_S_ is one of the largest fluxes in the terrestrial carbon cycle but its magnitude and dynamics remain poorly constrained.

We hypothesized that boreal tree stress or mortality [12], [13] might be exerting a significant effect on the large-scale, high-latitude *R*_S_ flux, as belowground carbon allocation drops in weakening or dying trees. Such forest stress and mortality has been observed in both boreal North America [14], [15] and Eurasia [16], [17], as well as more broadly worldwide [18]. These events are most frequently attributed to drought stress [19] or insect attack [20], and can be observed as trends in the remotely-sensed Normalized Differenced Vegetation Index (NDVI), a measure of canopy greenness [21], [22], as well as the Enhanced Vegetation Index (EVI) [23]. Such severe stress events are associated with canopy defoliation and depletion of carbon reserves, delayed recovery of surviving individuals, and tree death [24], [25]. Because plant photosynthesis is the ultimate source of all ecosystem respiration, and forest soil respiration at large scales may be driven more by productivity than temperature [26], such events should also, in theory, be observable in *R*_S_ data.

More generally, climate changes appear to be observable in the extant published record of *R*_S_ fluxes [10], but how such large-scale changes interact to affect the major components of the high-latitude carbon cycle remains an open question [6]. To explore one aspect of this, we linked a global *R*_S_ database [27], NDVI or canopy greenness [22], [28] and gridded climate data using both machine-learning and classical statistical approaches. Our objectives were to analyze the relationship, if any, between forest ‘browning’ observed from satellites and large-scale patterns of annual *R*_S_, and to infer constraints that may be operating at high latitudes on this large carbon flux.

## Methods

### Soil Respiration, NDVI, and Ancillary Data

Observed soil surface CO_2_ flux, or soil respiration (*R*_S_, g C m^−2^ yr^−1^), was the primary response variable considered in this study. We used a recent version (20110224a, downloaded 24 February 2011 from http://code.google.com/p/srdb/) of a global soil respiration database [27]. The downloaded data were filtered to include only non-manipulated ecosystems (no agriculture or experimentally manipulated systems); positive *R*_S_ values; >50°N latitude; mean annual air temperature (1961–1990) of <2°C, following [10]; and measured using infrared gas analyzers or gas chromatography, relatively standardized techniques.

The primary independent data were Advanced Very High Resolution Radiometer-Normalized Difference Vegetation Index (AVHRR-NDVI, from http://glcf.umiacs.umd.edu/data/gimms/) data covering all land surfaces above 50°N, except the glaciated areas of Greenland. These NDVI measure ‘greenness,’ which at the pixel level declines (and ‘browning’ increases) as forests weaken and eventually die from biotic or abiotic stresses. These data were produced as part of the NASA Global Inventory, Monitoring and Modeling project (GIMMS version-G), spanned the years 1982–2008 and were relatively coarse in spatial (64 km^2^ cells) and temporal (15-day composite images) scales. GIMMS version-G data have been calibrated to account for orbital drift, cloud cover, sensor degradation, and the emission of volcanic aerosols [29], [30]. We transformed these data to a stereographic polar projection based on the Clarke 1866 spheroid, and summarized them at a variety of temporal scales: monthly; seasonal, including spring (mean of March and April), early summer (May, June), late summer (July, August), autumn (September, October), and winter (November-February); and annual (mean of the entire year).

A variety of ancillary data were included in the analysis. Time since disturbance (in years) was derived from the soil respiration database, above, with missing data assigned the median value (∼50 years) as recommended by [31]. (Excluding the missing data resulted in a significantly smaller data set, but did not change the disturbance-related results below.) Global climate data (“Monthly Mean Air Temperature (Global 1900–2008)” and “Monthly Total Precipitation (Global 1900–2008)”) sets were downloaded from http://climate.geog.udel.edu/~climate/; these data were used because of their spatial resolution and currency. Mean (1961–1990) values and climate anomalies were then computed as the year-specific temperature or precipitation value minus the mean value for that 0.5° grid cell. Global leaf area index (5″, from ECOCLIMAP [32]), grid area (0.5°, to derive a circumpolar flux from area-normalized predictions, from EOS-WEBSTER at http://eos-webster.sr.unh.edu/), nitrogen deposition (5°, from ORNL DAAC at http://webmap.ornl.gov/wcsdown/wcsdown.jsp?dg_id=830_2), a Thornwaite-based climate index [33], and the MODIS Vegetation Continuous Field (Collection 4, Version 3, from http://www.landcover.org/data/vcf/) were also used.

These data sets were matched spatially and temporally to the collected *R*_S_ studies using a nearest-neighbor algorithm. Temporally, each *R*_S_ observation was paired with climate and NDVI data from the year of that study as well as up to five years previously, i.e., a given *R*_S_ observation from year *t* was associated with temperature anomaly, precipitation anomaly, and NDVI (half-monthly, monthly, etc., as described above) data from year *t*, *t*-1, … *t*-5. This was done because multi-year carbon pools in northern ecosystems [34] may decouple observed carbon fluxes (e.g., tree growth) from ambient abiotic drivers [22], [35].

**Table 1:** Summary of variable importance in conditional inference random forest models.

Variable name	Rank	Models	Variable description
ndvi_jun4	1.4	5	NDVI, June, 4 years previous
ndvi_jun1	2.3	11	NDVI, June, previous year
ndvi_juna1	2.4	5	NDVI, first half of June, previous year
ndvi_maya3	3.7	3	NDVI, first half of May, 3 years previous
ndvi_sepa1	4.6	5	NDVI, first half of September, previous year
ndvi_esummer4	4.7	7	NDVI, early summer, 4 years previous
ndvi_esummer1	5.2	16	NDVI, early summer, previous year
ndvi_jun0	5.3	12	NDVI, June, previous year
ndvi_juna4	5.5	2	NDVI, first half of June, 4 years previous
ndvi_junb4	5.5	2	NDVI, second half of June, 4 years previous
ndvi_juna5	7.0	1	NDVI, first half of June, 5 years previous
ndvi_may3	8.1	7	NDVI, May, 3 years previous
ndvi_apr3	9.0	1	NDVI, April, 3 years previous
ndvi_auga2	9.3	4	NDVI, first half of August, 2 years previous
ndvi_lsummer5	9.3	4	NDVI, late summer, 5 years previous

Only the top 15 variables (out of 270 total potential predictors) are shown. Variables are ordered by the mean rank (from node purity) computed by the random forest algorithm; the third column gives number of models across which this mean was computed.

### Data Analysis

Two related machine-learning algorithms were used on the final, unified data set (105 observations and 288 variables, from 64 unique sites). The standard Random Forest algorithm [31], a nonparametric machine learning technique for classification and regression, is widely used for large-data analyses, and as a data-driven methodology makes no *a priori* theoretical assumptions about *R*_S_ drivers or behavior. The algorithm predicts by aggregating regression trees constructed using different random samples of the data, and choosing splits of the trees from subsets of the available predictors, which are randomly chosen at each node [31]. The use of random data and predictor subsets means that the full data set can be used and data need not be withheld for validation. The RF algorithm generally produces highly accurate and unbiased estimates and classification when potential predictors are drawn from the same scale or category, and it is particularly robust against overprediction for ‘m>n’ (more potential predictor variables than observations) data sets.

**Figure 1:**
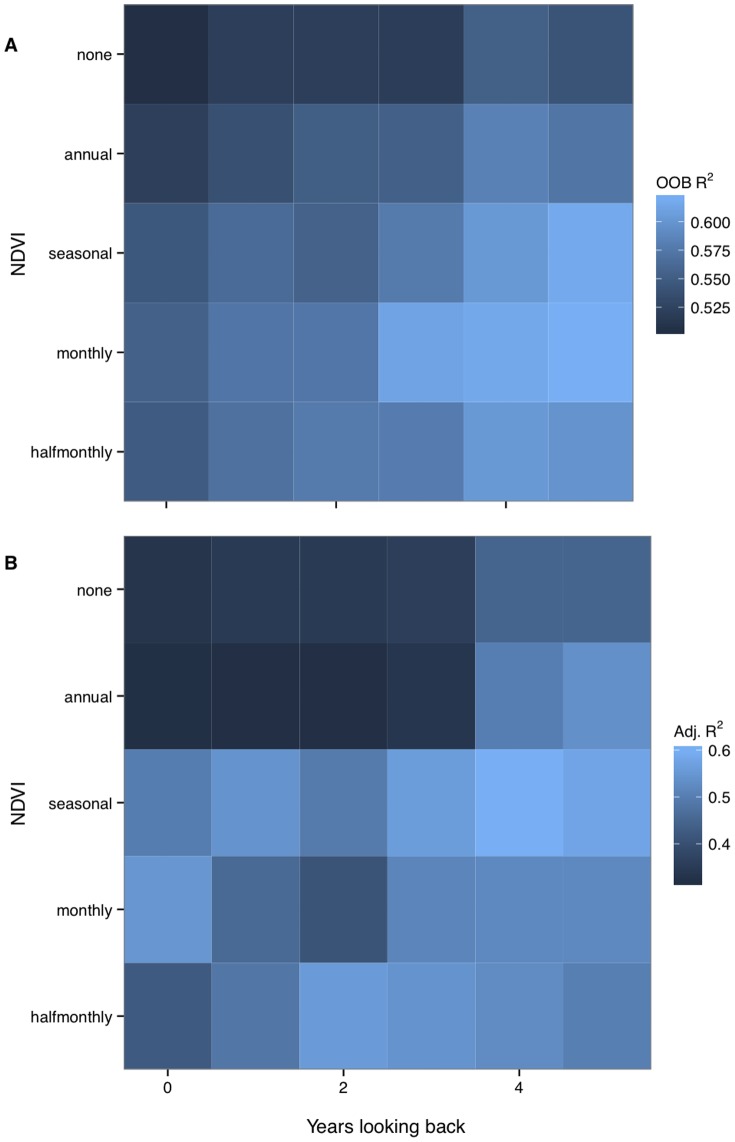
Summary of model performance in predicting high-latitude soil respiration. Data are shown by algorithm type (a: conditional inference Random Forest, CI-RF; b: ordinary least squares, OLS), level of NDVI detail available to the algorithm (none, and annual, seasonal, monthly, and half-monthly means), and number of years the algorithm was allowed to look into the past. Values given are bin midpoints; out-of-bag R^2^ for CI-RF (see Methods), and adjusted R^2^ for OLS.

Random Forest can be unreliable (exhibit biased variable selection), however, when potential predictor variables vary in measurement scale or categories [36]. For this reason we also used a conditional inference random forest (CI-RF) algorithm [37], the *cforest* routine in *party* package version 0.9-99991 in R [38]. This algorithm supports conditional inference trees [37] and aggregates using observation weights extracted from the trees [39]. Such conditional inference forests better handle variables of different types, and observations of different weights, than do trees generated using the original Breiman RF algorithm, although they do not entirely eliminate the preference for correlated predictors [37].

**Table 2:** Summary of the best-performing ordinary least squares (OLS) model.

Variable	Year	Estimate	SE	t	P	Signif.
(Intercept)		−16760	9687	−1.73	0.087	.
NDVI (early summer)	1	13.96	5.02	2.78	0.007	**
NDVI (late summer)	4	7.77	5.15	1.51	0.135	
NDVI (early summer)	0	6.85	4.81	1.43	0.158	
Air temperature	4	1.03	1.66	6.21	<0.001	***
NDVI (late summer)	1	−1.53	4.96	−3.08	0.003	**
Precipitation	0	0.70	0.34	2.05	0.043	*
Year		8.51	4.84	1.76	0.082	.
Air temperature	0	−55.19	22.81	−2.42	0.017	*
Mean annual precip.		−0.58	0.20	−2.90	0.005	**
NDVI (annual)	1	−14.43	6.31	−2.29	0.025	*
NDVI (fall)	0	9.21	3.35	2.75	0.007	**

Potential parameters of the best OLS model (RMSE = 156.9 g C m^−2^ yr^−1^ on 94 d.f., adjusted R^2^ = 0.61, P<0.001) were selected by the CI-RF algorithm before OLS was performed (see Methods and Table 1). Columns include variable included in OLS regression, year of data stream (0 = current year, 1 = previous year, etc.); OLS estimate and standard error (SE); t-value; P-value; and significance (“.” <0.1; “*” <0.05; “**” <0.01; “***” <0.001).

We allowed these algorithms to access varying amounts of NDVI (from the original 15-day data, to monthly, seasonal, and annual means, to none at all) and previous-year information (‘lookback,’ from 0 to 5 years in the past). Importantly, each level tested included all previous coarser ones; for example, models using monthly NDVI data were also given seasonal and annual data, to see if the new level of detail resulted in significant model improvement. Because late-winter snow interferes with the satellite sensor, resulting in many missing values for this time period, we excluded December-April NDVI after extensive testing: none of these data was significant (i.e., ranked in the top 25 most important variables; cf. **Table 1**) in any tested *R*_S_ model, and their exclusion resulted in no decrease in model explanatory power. The RF and CI-RF routines were run with default settings (in particular, number of variables randomly selected at each node = 5, number of trees = 500) for all 30 models (5 levels of NDVI information times 6 levels of temporal lookback); we found that altering these parameters did not change the results in any meaningful way. The algorithms ranked all variables by importance. For CI-RF, we computed a pseudo-R^2^ following the original *randomForest* package, as 1-SS_TOT_/SS_ERR_, because the *party* package does not currently compute a true out-of-bag error rate.

**Figure 2:**
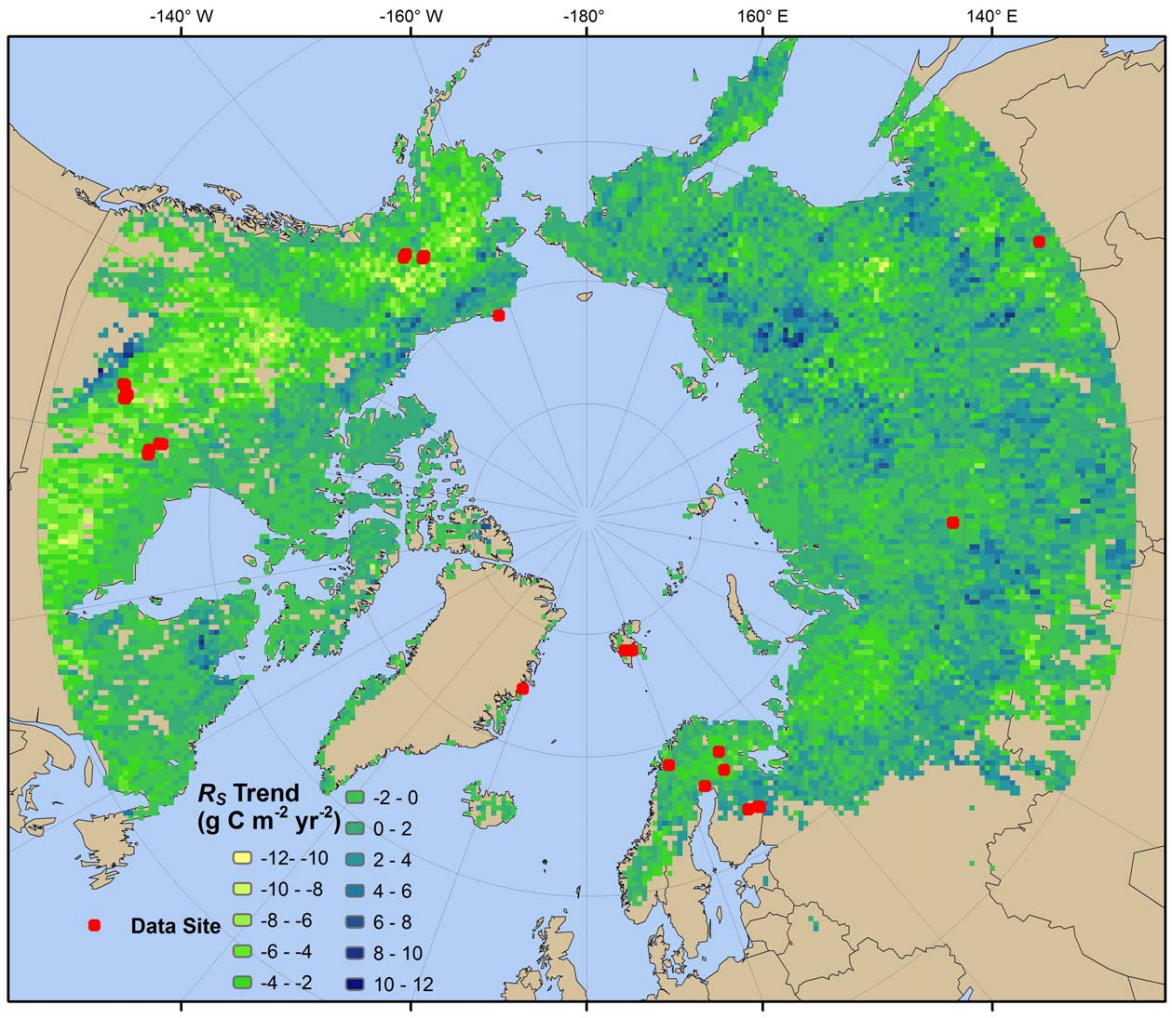
Spatial distribution of 1989–2008 soil respiration trends (*R*_S_, g C m^−2^ yr^−2^). Grid cells are colored by slope of *R*_S_ trend, computed based on the best fitting model (conditional-inference Random Forest, using monthly NDVI data up to 5 years previously) from Table 1. Field studies used in building the models, drawn from a global *R*_S_ database [27], are shown by overlaid points.

We also examined the effect of including the most important variables, as identified by the RF and CI-RF algorithms, into ordinary least squares (OLS) models, as OLS is a fundamental tool for analyzing sources of variance in many studies. For each of the 30 NDVI/lookback models we built ordinary least squares (OLS) models using the 18 most important variables identified by the machine-learning algorithms. The automated ‘step’ function in R removed and added model terms, starting from the complete formula identified by the RF (and CI-RF) analysis. Term selection was based on Akaike Information Criterion. For all analyses, observations were weighted by the years of observed data reported for each *R*_S_ data point, to account for studies that reported multi-year *R*_S_ means. OLS models were checked for influential outliers using a Cook’s distance threshold of 0.5 and refit, if necessary, after outlier removal.

**Figure 3:**
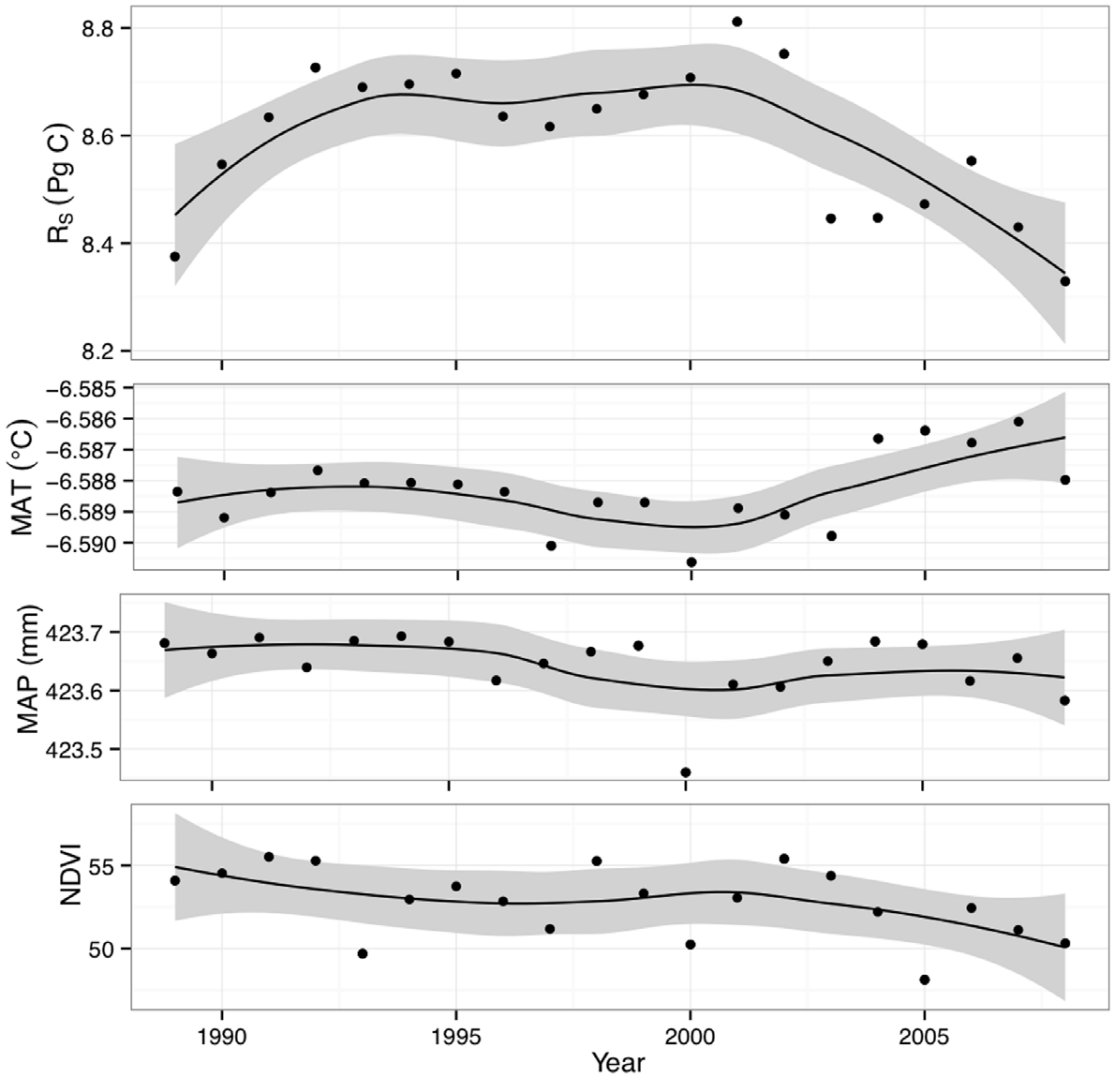
Predicted high-latitude soil respiration (*R*_S_), by year, with main driver variables. Panels show, from top, *R*_S_ predicted flux; mean annual temperature (MAT); mean annual precipitation (MAP); and previous-June canopy greenness (NDVI, unitless). *R*_S_ points show integrated result of the best-performing Random Forest model; to highlight trend, a loess smoother is shown by the dark line. Smoother errors (gray regions) were computed as the least-squares error on locally weighted scatterplot smoothing.

### Circumpolar Modeling

The best-performing (based on pseudo-R^2^) model was used to predict *R*_S_ fluxes across the circumpolar region. A circumpolar 0.5° grid was used, with grid cells matched to all required climate, NDVI, and ancillary data. Predicted fluxes for years 1989–2008–roughly the period of methodologically standardized and published *R*_S_ measurements [10]–were calculated using the cell area data and summed to produce a global high-latitude flux for boreal and Arctic (>50°N, mean annual air temperature <2°C) cells. A nonparametric Mann-Kendall test was used to test for temporal trends in the model output, and D’Agostino’s *K* ^2^ goodness-of-fit [40] to test for skew or departures from normality. All analyses were performed using R 2.15.1 [38].

**Figure 4:**
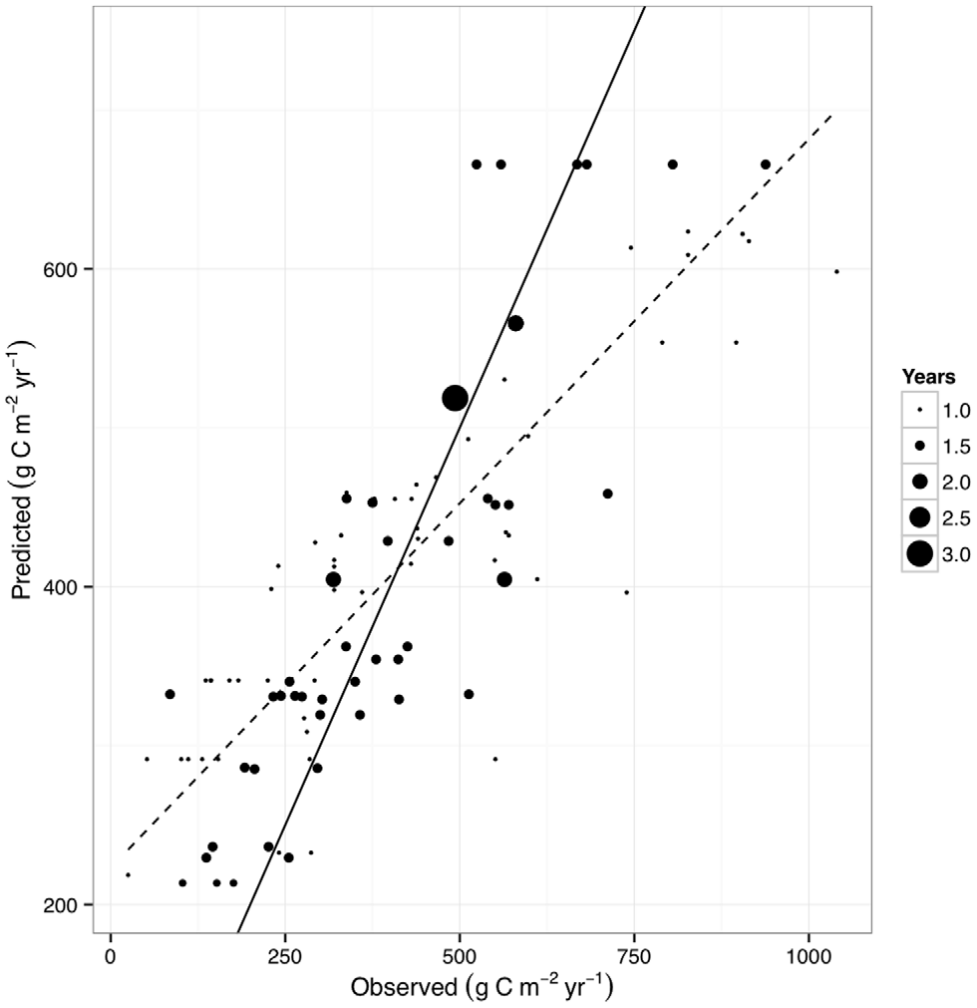
Observed versus predicted soil respiration (g C m^−2^ yr^−1^) for the best-performing linear model summarized in Table 1. Solid line shows 1∶1, dashed line (with grey error region) the relationship between observed and predicted values. Point size indicates number of years reported by each study (cf. Figure 2), and was used as a weighting factor in all analyses.

## Results

The two machine-learning models accounted for 50–62% of the observed variability for 105 annual *R*_S_ observations at high latitudes. When allowed to use more-detailed NDVI data, and look back further into the past (i.e., consider previous-year conditions to explain current *R*_S_) the models’ performance improved (**Figure 1**). The best-performing model (a CI-RF type, root mean square error RMSE = 139.9 g C m^−2^ yr^−1^) used monthly NDVI and up to five previous years’ NDVI/climate data; this was also identified as the best model by the classical RF algorithm. OLS models built using the most important variables from the machine-learning analyses showed a dramatic improvement, with explained variability almost doubling from 33% (no NDVI, only current-year data) to 61% (seasonal NDVI, up to four years ‘lookback’ allowed). The best-performing OLS model is summarized in **Table 2**. For all algorithms, the use of half-monthly NDVI did not improve model performance relative to mean monthly NDVI.

Previous-year NDVI–in particular, early-summer greenness–was generally the most important *R*_S_ predictor. Nine of the top ten CI-RF variables, and five of the top ten RF ones, were NDVI in early summer (May, June, July) in years before the *R*_S_ measurement, especially the previous year (Table 1). The best-performing models also followed this pattern, with previous-year June NDVI the best predictor and previous-year temperature the best non-NDVI predictor. A number of potential explanatory variables were almost never highly ranked, including nitrogen deposition, time since disturbance, leaf area index, and percent tree cover. As in a previous study [10], mean annual air temperature was negatively associated with *R*_S_–i.e., warmer years were consistently associated with lower respiration at high latitudes. In summary, NDVI proved a far better *R*_S_ predictor than any other type of variable; previous-year data almost always outperformed current-year data; and early-summer NDVI was the single best predictor across a large number of models.

The results of extrapolating *R*_S_ across the circumpolar boreal region based on NDVI and climate data for the 1989–2008 period are shown spatially in **Figure 2**, and the integrated circumpolar flux in **Figure 3**. Predicted *R*_S_ values ranged from 212–646 g C m^−2^ yr^−1^, with a mean±s.d. of 348±102 g C m^−2^ yr^−1^; in comparison, Arctic and boreal data average 109±109 and 383±228 g C m^−2^ yr^−1^, respectively, in the *R*_S_ database used here [27].

The model did not predict extremely low (<100 g C m^−2^ yr^−1^) *R*_S_ values observed at some Arctic sites, probably due both to the paucity of observed *R*_S_ data at these extreme latitudes, and the presence of late-lying snow that interferes with the satellite sensor. High observed *R*_S_ values also tended to be underpredicted (**Figure 4**). The mean predicted *R*_S_ integrated over the entire study area was 8.6 Pg C yr^−1^, ∼9% of the global flux [10], and declined (0.04 Pg yr^−2^, Mann-Kendall tau = -0.511, *P* = 0.049) over the last ten years of the study period. Large areas of declining *R*_S_ in western North America (yellow patches in **Figure 2**) drove the circumpolar slowdown in the model output.

## Discussion

The dominance of lag effects–in previous-year NDVI and air temperature–in this analysis is consistent with both theory and observations. Short-turnaround, labile C comprises a significant component of ecosystem C fluxes [41], while field experiments have shown a lack of correlation between boreal tree ring width increment and net ecosystem exchange [35], while ring width and NDVI are only inconsistently correlated in high-latitude forests [42], [43]. This suggests that multi-year C pools play a significant role in buffering ecosystem carbon fluxes from changing abiotic drivers. Lags between *R*_S_ and its drivers (soil temperature and gross primary production) of up to 88 days were shown by Vargas et al. [44], but we are unaware of previous studies documenting multi-year lag effects. We note that RF and CI-RF models using no current-year data at all–simply previous-year NDVI, air temperature, and precipitation–explains ∼60% (RMSE = 140.9 g C m^−2^ yr^−1^) of observed *R*_S_ variability, i.e., adding current-year data yields very little improvement in model performance.

What mechanisms would link increases in temperature with declines in *R*_S_, as observed here? Largely following the logic of Peng et al. [14], we hypothesize that drought and water stress engender hydraulic failure and inability to maintain carbon balance (i.e., starvation) [25]. The dominant sources of *R*_S_ are root (autotrophic) and microbial (heterotrophic) respiration, and both are affected–albeit at different temporal lags–by changes in the photosynthate supply [45]. The resulting declines in belowground tree respiration and root exudates then depress the *R*_S_ flux as measured at the soil surface.

Such a mechanism would be consistent with other studies performed at a variety of scales. Drought has deleterious effects on CO_2_ uptake [46], and has been shown to reduce *R*_S_ in field studies [47]. Tree mortality in western boreal North America has increased [14], and field studies have observed aspen and white spruce stress and dieback in North America has been linked to moisture indices [12]. Silva et al. [15] reported that temperate and boreal trees in Ontario, Canada, exhibited widespread growth decline consistent with warming-induced stress, in spite of increases in water use efficiency over the last half-century. At larger scales, FLUXNET analyses have inferred significant drought effects on ecosystem carbon cycling [48], [49], and productivity (for which NDVI, in this study, is a proxy) has been shown to be more important than temperature in determining landscale-level *R*_S_ [26]. Finally, remote sensing analyses suggest that changes in annual temperature and precipitation across North America are negatively affecting forest resilience as measured using the MODIS Enhanced Vegetation Index [23]. The use of previous-year NDVI in this study is thus a significant strength, as it provides an integrated signal of forest canopy stress tightly linked with the photosynthates stored for the following year’s growth and respiration.

There are other possible mechanisms to explain a putative *R*_S_ slowdown: climate changes might enable more pathogen and pest outbreaks in drought-weakened trees [25] or increase freeze-thaw events [12], for example, resulting in tree death, lower NDVI and lower *R*_S_. Increased nitrogen deposition could also be depressing forest *R*_S_, as has been shown to occur in temperate forests [50], although most North American boreal and Arctic sites are considered nitrogen-limited. We found no association between *R*_S_ and nitrogen deposition and thus a water-related mechanism, as laid out above, seems more consistent with the available data.

It is not surprising that time since disturbance exerted no effect on *R*_S_ in this analysis. This is not to say that disturbance exerts no effect: plant productivity exerts a dominant role on *R*_S_ [26], and fire in particular plays an important role in many high-latitude forests [51], altering *R*_S_ by killing plants, increasing litter inputs, changing soil moisture conditions, and increasing the active layer depth [52]. Disturbances can also cause soil C losses (via *R*_S_) so large that sites become multi-year carbon sources [26]. The time-since-disturbance variable may simply not have added any extra information, however, given the strong NDVI effect found in this analysis and the fact that NDVI and time since disturbance tend to be well-correlated for several decades following disturbance [53]. In addition, while post-disturbance *R*_S_ changes may be visible in meta-analyses [54] and syntheses [26], many studies have observed inconsistent or invariant ecosystem respiration [55] and *R*_S_ [56] in the decades after disturbance. Finally, relatively few *R*_S_ studies have been performed in post-disturbance forest and tundra [27].

This analysis has a number of limitations. First, although we used two more years of data (observations published 2009–2010) than a previous *R*_S_ meta-analysis [10], these results are based on only 105 annual flux measurements spread across a large (∼24×10^6^ km^2^) circumpolar region. The possibility of a type I (false positive) error remains [10] significant: future data may resolve the curiosity of high-latitude *R*_S_ changes not being positively correlated with air temperature increases. Second, the *R*_S_ data used here are dominated by well-drained, boreal, upland sites, reflecting an imbalance in the published literature [27]. But the respiration of peatland and permafrost ecosystems–which store an outsized fraction of global soil organic carbon–may change in different ways than a simple temperature- and NDVI-based model would predict, driven by species shifts, permafrost thaw, and increasing peat oxygenation. Tundra ecosystems will also likely respond differently to warming than will boreal forests, as processes such as warming-induced thermokarst and woody plant encroachment may increase plant productivity [6], [57], [58]. Finally, *R*_S_ cannot by itself be used to infer carbon balance, as ecosystem carbon balance is driven by the balance between net primary production and heterotrophic respiration from snags, woody debris, and soil. Few such comprehensive data are available at high latitudes, however.

### Conclusions

This study has shown that remotely-sensed NDVI and climate data explain a large fraction of the variability of *R*_S_, the dominant component of ecosystem respiration, at high latitudes. Combining large-scale observations (NDVI) and a compilation of small-scale observations (*R*_S_) allowed us to show that lag effects imply multi-year carbon pools exerting significant large-scale effects, to the point that no current-year data are needed (at this scale) to predict total *R*_S_ in a given year. Finally, we suggest that high-latitude *R*_S_ has declined significantly over the last ten years, as warmer summers stress some northern ecosystems, in particular the boreal forests that constitute most of the data used here [46]; we caution that tundra ecosystems may respond very differently. Although we cannot prove causality between the observed NDVI and *R*_S_ data, such an effect would be consistent with other recent studies (e.g., [59]). Because the boreal and Arctic carbon cycles may exert strong global climate feedbacks [6], the question of whether this decline is truly a symptom of water stress and forest mortality deserves further exploration.
